# Triparental origin of triploid onion, *Allium* × *cornutum* (Clementi ex Visiani, 1842), as evidenced by molecular, phylogenetic and cytogenetic analyses

**DOI:** 10.1186/1471-2229-14-24

**Published:** 2014-01-13

**Authors:** Željana Fredotović, Ivica Šamanić, Hanna Weiss-Schneeweiss, Juraj Kamenjarin, Tae-Soo Jang, Jasna Puizina

**Affiliations:** 1 Department of Biology, University of Split, Faculty of Science, Teslina 12, 21000 Split, Croatia; 2 Department of Systematic and Evolutionary Botany, University of Vienna, Rennweg 14, A-1030 Vienna, Austria

**Keywords:** *Allium* × *cornutum*, *Allium cepa*, *Allium roylei*, *Allium pskemense*, Triparental hybrid, Triploid, Fluorescence *in situ* hybridisation (FISH), ITS1-5.8S-ITS2, 5S rDNA non-transcribed spacer (NTS), Genomic *in situ* hybridisation (GISH)

## Abstract

**Background:**

Reconstruction of the parental origins of cultivated plants from wild relatives, especially after long periods of domestication, is not a trivial task. However, recent advances in molecular phylogenetics, among other approaches, have proved to be very informative in analyses of the origin and evolution of polyploid genomes. An established minor garden crop, triploid onion *Allium × cornutum* (Clementi ex Visiani, 1842) (2*n* = 3*x* = 24), is widespread in southeastern Asia and Europe. Our previous cytogenetic analyses confirmed its highly heterozygous karyotype and indicated its possible complex triparental genome origin. *Allium cepa* L. and *Allium roylei* Stearn were suggested as two putative parental species of *A.* × *cornutum*, whereas the third parental species remained hitherto unknown.

**Results:**

Here we report the phylogenetic analyses of the internal transcribed spacers ITS1-5.8S-ITS2 of 35S rDNA and the non-transcribed spacer (NTS) region of 5S rDNA of *A*. × *cornutum* and its relatives of the section *Cepa*. Both ITS and NTS sequence data revealed intra-individual variation in triploid onion, and these data clustered into the three main clades, each with high sequence homology to one of three other species of section *Cepa*: *A. cepa*, *A. roylei*, and unexpectedly, the wild Asian species *Allium pskemense* B. Fedtsh. *Allium pskemense* is therefore inferred to be the third, so far unknown, putative parental species of triploid onion *Allium × cornutum*. The 35S and 5S rRNA genes were found to be localised on somatic chromosomes of *A*. × *cornutum* and its putative parental species by double fluorescent *in situ* hybridisation (FISH). The localisation of 35S and 5S rDNA in *A.* × *cornutum* chromosomes corresponded to their respective positions in the three putative parental species, *A. cepa*, *A. pskemense*, and *A. roylei.* GISH (genomic *in situ* hybridisation) using DNA of the three putative parental diploids corroborated the results of the phylogenetic study.

**Conclusions:**

The combined molecular, phylogenetic and cytogenetic data obtained in this study provided evidence for a unique triparental origin of triploid onion *A.* × *cornutum* with three putative parental species, *A. cepa*, *A. pskemense*, and *A. roylei.*

## Background

Polyploidy and hybridisation are regarded as important processes accompanying and contributing to plant diversification and speciation. Allopolyploidy, which involves both of these processes, is regarded as a particularly important driving force of plant evolution. Inferring the parental origin of allopolyploids is not a trivial task, particularly after long periods of hybrid domestication. Recent technological advances and the common use of DNA sequence data for phylogenetic reconstructions revolutionised this field and enabled the identification of the parental taxa of many allopolyploids [1]. Most of the established allopolyploids are of biparental origin. There are very few reported cases of triparental polyploids, although well-known examples include common wheat, *Triticum aestivum*, which is allohexaploid and of triparental origin [2] and tetraploid Damask roses [3]. Most of the known allopolyploids are established on even-ploidy levels with very few consistently odd-ploidy level taxa persisting in nature (e.g., pentaploid *Rosa canina*) [4,5]).

Triparental odd-ploidy plant allopolyploids have rarely been found. Previous analyses of established and vegetatively propagating triploid *Allium × cornutum* (Clementi ex Visiani, 1842) suggested that it is one of the rare cases of allotriploid of triparental origin, and two parental taxa were suggested based on cytogenetic analyses of chromosome complements using GISH (genomic *in situ* hybridisation) [6]. The third parental taxon remained unknown. Triploid onion *Allium* × *cornutum* is traditionally cultivated in southern and coastal Croatia under the name ‘Ljutika’ (meaning ‘shallot’ in Croatian), and it is very popular as a spice and condiment due to its tasty bulbs and leaves [7]. Similar triploid onions are cultivated as garden crops in southeastern Asia, Europe, and other parts of the world [8-12]. Triploid onion was first described as *Allium cepa* L. var. *viviparum* (Metzg). Alef. [9,13,14]; however, Friesen < Klaas [11] suggested that this name is connected with the other viviparous onion *A*. × *proliferum*, and they proposed the use of the name *Allium cornutum* Clementi ex Visiani [15], which is the only name that is unambiguously connected with triploid onion [10,16]. However, taking into account its hybrid origin, the name was modified to *Allium* × *cornutum*[11]. This name was first used by Visiani [15] for a Dalmatian bulbiferous taxon that was observed for the first time on rocks in Dubrovnik [16].

In contrast to most flowering alliums in which the leaves start to senesce during flowering, triploid onions are perennials; their leaves remain green throughout the entire year. The plants are sterile and propagate vegetatively by underground bulbs and bulbils formed from the inflorescence. Phenotypically, triploid onions closely resemble *A. cepa*, and it is sometimes difficult to distinguish between these two types before the development of inflorescences. Inflorescences of triploid onion are composed of fewer and slightly larger flowers than *A. cepa* inflorescences. During inflorescence maturation, small reproductive bulbils are formed, and the flowers gradually wilt. Mature inflorescence may contain 20–30 small reproductive bulbils. Other reliable characters that allow the distinction between triploid shallot and *A. cepa* are the morphological features of the underground bulbs, which are elongated and pear-shaped in triploid onions; 10–20 or more individual bulbs usually grow together. The triploid onion leaves are intermediate in shape between semicircular and round, and the stalk that bears the inflorescence is only slightly flattened at the bottom, whereas that of *A. cepa* is inflated at the base [17].

The karyotype of *A.* × *cornutum* consists of 2*n* = 3*x* = 24 chromosomes [8,9]. The homology among the chromosomes is weak and occasional, and it is very difficult, if not impossible, to identify homologous chromosomes [6]. The most common meiotic chromosome associations of *A*. × *cornutum* are heterotrivalents, which suggests at least partial homology of the three genomes [14]. Additionally, the frequent occurrence of complex multivalents was observed, suggesting that the triploid karyotype might be due to translocations and other chromosomal rearrangements during evolution [14]. Mapping of the constitutive heterochromatin in *A.* × *cornutum* chromosomes by Giemsa C-banding demonstrated its hybrid genome structure with only one set of eight chromosomes shown to carry the heterochromatic markers typical of *A. cepa*[9]. Previously, triploid viviparous onions were speculated to be either of an allotriploid (AAB) [8] or segmental allotriploid (AA’A”) origin [18]. Several independent molecular studies pointed to *Allium cepa* as one of putative parental species of *A*. × *cornutum* (RFLP analysis of the chloroplast DNA and nuclear rDNA [19-21], isozyme analysis [10], and analysis of RAPD molecular markers [12]).

To determine the *A.* × *cornutum* origin, genomic *in situ* hybridisation (GISH) was applied [6,11]. Friesen < Klaas [11] confirmed *A. cepa* as a parental species of the triploid onion and concluded that the majority of DNA and chromosomes of *A.* × *cornutum* originated from *A. cepa.* Puizina *et al.*[6] showed that the genomic DNA of *A. cepa* and *A. roylei* each only or predominantly labelled only one chromosome set (eight chromosomes with genomic DNA of C – A. *cepa* and eight with R – A. *roylei*). The remaining chromosomes of the triploid karyotype were not labelled (or only partially and weakly labelled) by these two genomic probes. These GISH results provided the first indication that triploid onion might be of complex triparental origin.

Further progress in the identification of the parental species of *A.* × *cornutum,* an ‘enigmatic plant’ [12], was hampered by the lack of information on the phylogenetic relationships between common onion and its wild relatives (section *Cepa* of genus *Allium*). More recently, ITS sequences (internal transcribed spacers 1 and 2 of the 18S-5.8S-26S rDNA) of a large number of common onion relatives originating from Central Asia were deposited in GenBank [22-27] together with the sequences of the NTS (non-transcribed spacer) of 5S rDNA [28,29].

ITS sequences have frequently been used as a first-choice marker for inferring the phylogenetic relationships between various wild plant groups and particularly for inferring the origins of diploid and polyploid hybrids (e.g., [30-41]). ITS 1 and 2 are parts of the 18S-5.8S-26S nuclear ribosomal DNA, which is present in each eukaryotic genome in high copy number as tandem repeats in one to many loci per haploid genome [42,43]. rDNA units are prone to homogenisation via unequal crossing over and/or gene conversion [29,42]. Parental rDNA copies in hybrid organisms might evolve in various ways: (1) two parental rDNA types can potentially be retained, evolve independently, and provide direct evidence for historical hybridisation with or without polyploidisation (diploid homoploid hybrids vs. allopolyploids); (2) only one parental rDNA type might be retained in the genome of a hybrid, which typically can be achieved either by the conversion of all rDNA types towards one parental genome rDNA, or alternatively, rDNA of one parental genome might be removed from the hybrid genome; (3) hybrids might evolve new types of rDNA units that might (or might not) represent combinations of different parental rDNA units (reviewed in [44,45]).

Another subfamily of rRNA genes encompasses 5S rDNA repeats that are arranged in long tandem arrays in one to several loci in the genome. The 5S rDNA unit consists of a coding region (gene) that is approximately 120 bp in length and a non-transcribed spacer (NTSs), which in plants varies in length and base composition from approximately 100 to more than 700 bp [45,46]. The coding regions of 5S RNA are highly conserved, whereas NTS evolves much more rapidly. A high rate of base substitution of NTS in some plant groups qualifies this region as highly informative for molecular phylogenetic analyses and often aids in the identification of parental taxa of diploid and polyploid hybrids [1,46,47]. 5S rDNA does not undergo homogenisation, and unless physically deleted from chromosomes, all types of parental repeats can be detected in hybrids [1,36,37,48]. A lack of efficient homogenisation leads to considerable sequence heterogeneity among 5S rDNA spacer regions within the individual arrays, which has been reported in several plant groups [1,49-51].

In this paper, we infer the parental origin of allotriploid *Allium* × *cornutum* using molecular phylogenetic analyses of internal transcribed spacers (ITS1-5.8S-ITS2) of 35S rDNA and the non-transcribed spacer (NTS) of the 5S rDNA. The positions of these two classes of ribosomal genes have also been established in the somatic chromosomes of *A. × cornutum*, and the putative parental species were inferred from phylogenetic analyses (*A. pskemense*, *A. roylei*, and *A. cepa*). The triparental origin of *A.* × *cornutum* was confirmed using the genomic *in situ* hybridisation (GISH) technique. The newly obtained data are discussed in light of previously published data on genome origin, structure, and evolution of the triploid onion *A.* × *cornutum.*

## Results

### Molecular phylogenetic analysis of ITS and NTS sequences

The length of the ITS1-5.8S-ITS2 region in the four analysed *A.* ×*cornutum* individuals (each representing a different population; Table 1) ranged from 627 to 642 bps. The final ITS alignment of 48 sequences (clones) of *A. × cornutum* had 583 constant characters; 17 were parsimony-uninformative, and 27 were parsimony-informative. In total, 19 distinct ribotypes were found with the most frequent ribotype (denoted by the GenBank accession number KC783412) represented by 24 clones originating from all four individuals (Table 1). The number of variable characters was 18 in the ITS1, four in the 5.8S rRNA gene, and 22 in the ITS2 region (Additional file 1: Figure S1).

**Table 1:** **GenBank accession numbers for the ITS1-5.8S-ITS2 and NTS-5S rRNA sequences of A. × *cornutum *** **obtained in this study**

	**ITS1-5.8S-ITS2**			
KC783412	Dubrava_8	Hvar_2	Kastela_1	Vis_5
	Dubrava_15	Hvar_3	Kastela_2	Vis_10
	Dubrava_16	Hvar_4	Kastela_5	Vis_11
	Dubrava_18		Kastela_8	Vis_12
	Dubrava_22		Kastela_11	Vis_14
	Dubrava_23		Kastela_12	Vis_19
				Vis_20
				Vis_27
				Vis_28
KC783413	Dubrava_14			
KC783414	Dubrava_19	Hvar_10		
KC783415	Dubrava_23		Kastela_7	
KC783416	Dubrava_31		Kastela_6	
KC783417		Hvar_1		
KC783418		Hvar_8		
KC783419		Hvar_12		
KC783420		Hvar_17		
KC783421		Hvar_18		
KC783422				Vis_15
KC783423				Vis_16
KC783424				Vis_22
KC783425				Vis_26
KC783426				Vis_29
KC783427	Dubrava_10			
KC783428	Dubrava_12			
	Dubrava_30			
KC783429	Dubrava_13	Hvar_20		
	Dubrava_17			
KC783430		Hvar_21		
Total:	15	11	8	14
	**NTS-5S**			
KC794504	Dubrava_28			
KC794505				Vis_30
KC794506	Dubrava_36			
KC794507		Hvar_23		
KC794508		Hvar_26		
KC794509		Hvar_28		
KC794510	Dubrava_26			
KC794511	Dubrava_38			
KC794512				Vis_33
KC794513				Vis_39
KC794514	Dubrava_37			
KC794515				Vis_38
KC794516				Vis_40
KC794517	Dubrava_35			
KC794518				Vis_23
KC794519				Vis_31
KC794520		Hvar_15		
KC794521		Hvar_22		
KC794522		Hvar_13		
Total:	6	6	-	7

The plant material was collected from four different locations in Croatia: Dubrava, Hvar, Kastela, and Vis.

**Figure 1:**
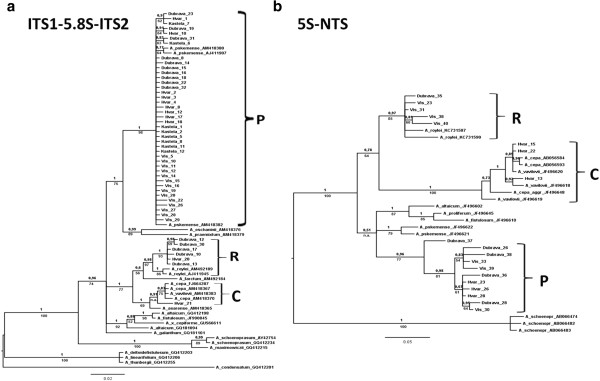
**Phylogenetic trees resulting from a Bayesian analysis of: a) the nuclear internal transcribed spacer (ITS) of *A*. × *cornutum *and the presumptive parental species *A. pskemense, A. roylei, A. cepa *and other species of the section Cepa; b) the non-transcribed spacer (NTS) of the 5S rDNA sequences of *A. *× *cornutum *and the presumptive parental species *A. pskemense, A. roylei, A. cepa *and other species of section *Cepa.*** The numbers above the branches depict Bayesian posterior probabilities, and the numbers below the branches indicate bootstrap support values from Maximum likelihood analysis (in the case of nodes not supported by all methods, the respective missing support values are indicated by ‘n.a.’). The bar indicates substitutions/site.

The sequences of the ITS regions of *A. × cornutum* were aligned to other ITS sequence data for *Allium* species of section *Cepa* available in GenBank (see Table 2 for the details). Three distinct clades of ITS1-5.8S-ITS2 sequences were identified within the *A. × cornutum* genome (Figure 1a, Additional file 1: Figure S1). Most of the sequences (41 sequences) formed one large clade, which also included ITS sequences of the wild Asian species *A. pskemense* and has therefore been designated xas clade P (“*pskemense*”-type). A second clade, containing six ITS sequences of *A. × cornutum*, grouped with the sequences of *A. roylei* and has been designated as clade R (“*roylei*”-type). One sequence of *A. ×cornutum* (from an individual from Hvar) showed similarity to *A. cepa* and *A. vavilovii* sequences, and hence, the whole clade was designated as clade C (“*cepa*”-type) (Figure 1a). The seven *A. × cornutum* ITS sequences consisting of clades R and C had a clearly distinguishable 13 base (CTGTAAACATACT) insertion in the ITS2 region, which is shared by both *A. cepa* and *A. roylei* but absent in *A. pskemense* (Additional file 1: Figure S1).

**Table 2:** List of taxa, GenBank accession numbers, and references for the previously published sequences used in this study

	**GenBank accession number (reference)**			
	**ITS1-5.8S-ITS2**		**NTS-5S**	
*Allium pskemense*	AM418380	(Gurushidze et al. 2007) [22]	JF496621	(Son et al. 2012) [28]
	AM418382	(Gurushidze et al. 2007)	JF496622	(Son et al. 2012)
	AJ411907	(Friesen et al. 2006) [23]		
*Alllium roylei*	AJ411945	(Friesen et al. 2006)	KC731587	This study
	AM492189	(Gurushidze et al. 2007)	KC731590	This study
*Allium cepa*	FJ664287	(Hirschegger et al. 2010) [24]	AB056584	(Shibata and Hizume 2002) [29]
	AM418367	(Gurushidze et al. 2007)	AB056593	(Shibata and Hizume 2002)
	AM418370	(Gurushidze et al. 2007)		
*Allium vavilovii*	AM418383	(Gurushidze et al. 2007)	JF496618	(Son et al. 2012)
			JF496619	(Son et al. 2012)
			JF496620	(Son et al. 2012)
*Allium cepa* var. *aggregatum*			JF496648	(Son et al. 2012)
*Allium oschaninii*	AM418376	(Gurushidze et al. 2007)		
*Allium praemixtum*	AM418379	(Gurushidze et al. 2007)		
*Allium farctum*	AM492184	(Gurushidze et al. 2007)		
*Allium asarense*	AM418365	(Gurushidze et al. 2007)		
*Allium altaicum*	GQ412198 GQ181094	(Jang et al. unpublished) (Li et al. 2010) [25]	JF496602	(Son et al. 2012)
*Allium fistulosum*	JF990845	(Guenaoui et al. 2013) [26]	JF496610	(Son et al. 2012)
*Allium x cepiforme*	GU566611	(Li et al. 2010)		
*Allium galanthum*	GQ181101	(Li et al. 2010)		
*Allium x proliferum*			JF496645	(Son et al. 2012)
*Allium schoenoprasum*	AY427547	(Ricroch et .al. 2005) [27]	AB066483	(Shibata and Hizume 2002)
	GQ412234	Jang et al. unpublished	AB066482	(Shibata and Hizume 2002)
			AB066474	(Shibata and Hizume 2002)
*Allium maximowiczii*	GQ412215	Jang et al. unpublished		
*Allium deltoidefistulosum*	GQ412203	Jang et al. unpublished		
*Allium linearifolium*	GQ412206	Jang et al. unpublished		
*Allium thunbergii*	GQ412255	Jang et al. unpublished		
*Allium condensatum*	GQ412201	Jang et al. unpublished		

The exceptions are the sequences for the NTS-5S genes of *A. roylei*, which were obtained in the present study.

The presence of different ITS1-5.8S-ITS2 rDNA repeat types within the *A*. × *cornutum* genome and their genetic similarity to their putative parental species *A. pskemense*, *A. roylei*, and *A. cepa* was confirmed by phylogenetic analysis. The phylogenetic algorithms Maximum-Likelihood (ML) and Bayesian Inference (BI) resulted in nearly the same tree topology. Only the BI tree is shown (Figure 1a), which summarises the topology and posterior probabilities (PP) from BI plus bootstrap support (BS) from the ML analysis. All the ITS sequences of *A. × cornutum* clustered into the three clades: P, R and C (Figure 1a). The clades P and R were both strongly supported by both analyses (clade P: 1.0 PP, 98% BS; clade R: 0.98 PP, 97% BS). The clade C containing a single *A.* × *cornutum* sequence (Hvar 21) and sequences from *A. cepa, A. vavilovii* and *A. asarense* was less strongly supported (1.0 PP; 69% BS).

To analyse the variability of non-transcribed spacer (NTS) of the 5S rRNA genes in *A*. × *cornutum*, 19 clones were obtained from three *A. × cornutum* individuals (Table 1). The NTS region showed higher sequence variation than the ITS1 and 2 regions. Nearly all of the cloned sequences were unique. The length of the 5S rDNA unit including the non-transcribed spacer (NTS) region ranged from 339 to 346 bps. The conserved 5S rRNA coding region was excluded from further analyses. The NTS region comprised 224–231 characters, of which 148 were constant and 73 were variable (including 53 parsimony uninformative and 20 parsimony-informative characters). In total, 13 positions in the alignment included gaps (Additional file 2: Figure S2).

Phylogenetic analyses of NTS clones of *A. × cornutum* together with NTS sequences of their potential close relatives retrieved from GenBank (Table 2) supported the grouping of the *A*. × *cornutum* NTS sequences into the three well-supported clades (P, R and C; Figure 1b). The largest clade consisted of 11 sequences and grouped with the NTS sequences of *Allium pskemense, A. altaicum* and *A.* × *proliferum* (88–92% similarity), and this clade has been designated as clade P. The second largest clade (R) consisted of five NTS sequences of *A. × cornutum*, which grouped with *A. roylei* NTS regions (89% similarity)*.* The smallest clade (C) consisted of only three NTS sequences of *A. × cornutum*, which were 99% identical to the NTS sequences of *A. cepa* and *A. vavilovii.* The largest clade P, comprising 11 NTS sequences of *A. × cornutum*, has been clearly separated from the NTS sequences of its closest relatives, *A. altaicum*, *A.* ×*proliferum*, and *A. pskemense* (Figure 1b).

### Chromosomal localisation of 5S rDNA and 35S rDNA genes and genomic *in situ* hybridisation

**Figure 2:**
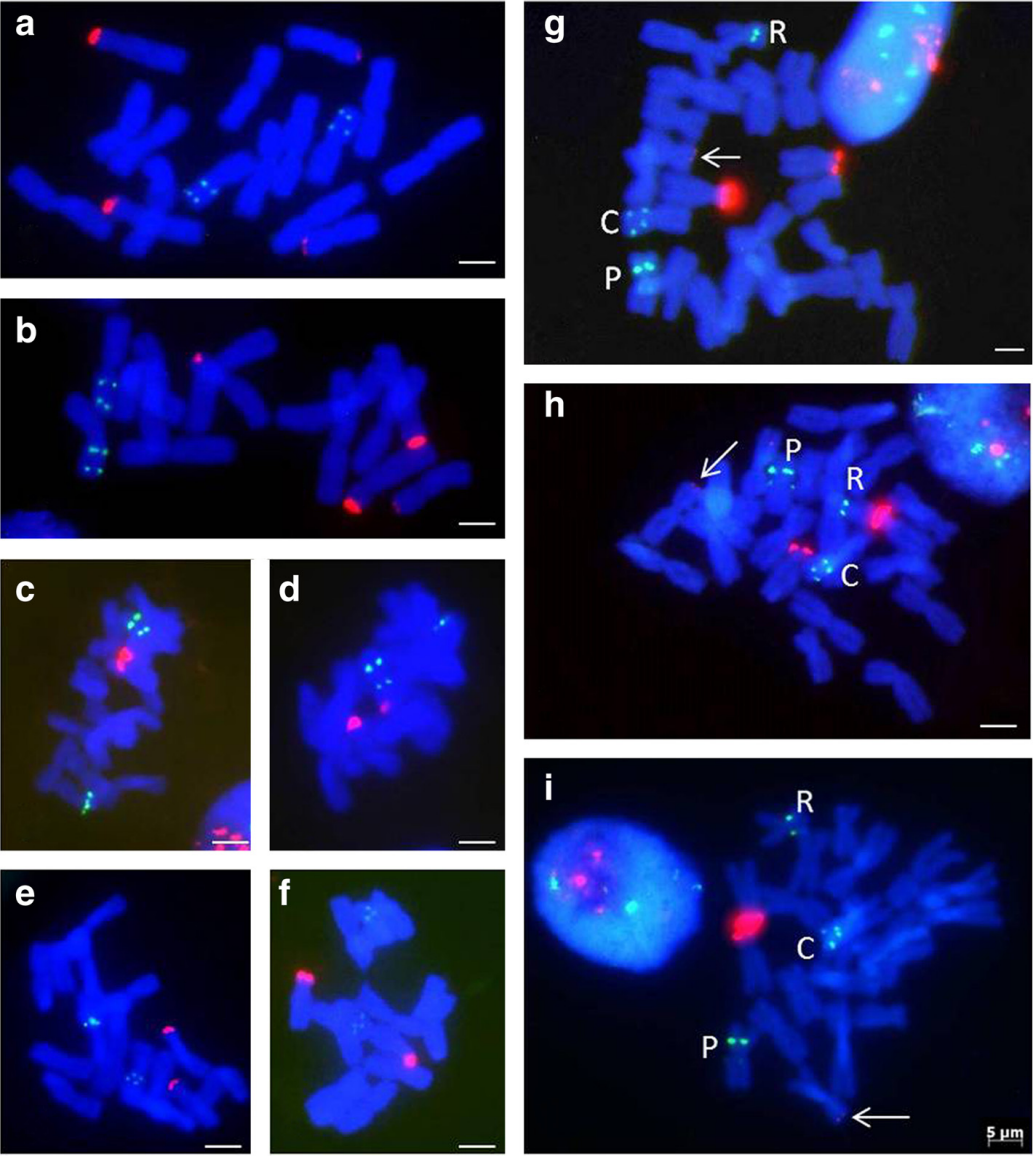
**rDNA mapping in the mitotic chromosomes of *Allium × cornutum *and the putative parental taxa (5S rDNA red, 35S rDNA green). (a, b)***Allium cepa*; **(c, d)***A. pskemense;***(e, f)***A. roylei*; **(g–i)***A.* × *cornutum*; Arrows in **g**, **h** and **i** indicate the smallest, barely visible 35S signal on the medium-sized metacentric chromosome originating from *A. cepa.* Scale bar = 10 μm.

The number and localisation of 5S and 35S rDNA loci were analysed in two clones of *Allium* × *cornutum* (Dubrava and Vis) and in three putative parental diploid taxa that were identified in phylogenetic analyses: *Allium cepa*, *A. pskemense* and *A. roylei. Allium cepa* possessed two loci of 35S rDNA in two satellite chromosome pairs and two 5S rDNA loci, both localised on the short arm of chromosome 7 (Figure 2a, b). The 35S rDNA loci differed in the intensity (and likely number of repeat copies). *A. pskemense* possessed one subterminal locus of 35S rDNA located on one satellite chromosome pair and at most two loci of 5S rDNA on chromosome 6 (Figure 2c, d). One larger locus of 5S rDNA was located interstitially in the short arm of chromosome 6 and the other, weaker locus was situated in the pericentric region of the long arm of the same chromosome. Two homologous chromosomes carrying 5S rDNA exhibited heterozygosity concerning the presence of a small 5S rDNA signal in the pericentromeric region of the long chromosome arm (Figure 2c,d). *A. roylei* possessed a single locus of 35S rDNA on a satellite-bearing chromosome pair (Figure 2e, f) and two 5S rDNA loci of similar size in the small metacentric chromosome 7. One of these 5S rDNA loci was localised close to the pericentric region, and the other was localised more internally within the same arm (Figure 2e,f).

In triploid *A*. × *cornutum*, two major subterminally localised signals of 35S rDNA were detected in the short arms of the two subtelocentric satellite chromosomes. A third minor 35S rDNA signal was detected on some spreads and was located in the subtelomeric region of one small sub-metacentric chromosome (white arrows in Figure 2g, h, i). The largest satellite chromosome (resembling the large NOR-bearing chromosome of *A. cepa*) lacked a 35S rDNA signal. Each of the three 35S rDNA signals differed in intensity and size, with the medium-sized chromosome carrying the strongest signal.

The 5S rRNA genes were detected in three differently sized chromosomes in *A. × cornutum* (Figure 2g, h, i). The largest chromosome carrying a 5S rDNA signal resembled the typical *A. cepa* chromosome with two 5S rDNA loci localised within the long arm of chromosome 7. The medium sized chromosome carrying 5S rDNA in *A. × cornutum* possessed two 5S rDNA loci, which differed in size and intensity, with the stronger signal positioned interstitially within the short arm and the weaker signal corresponding to the pericentromeric region of the long chromosome arm. This chromosome bears resemblance to the 5S rDNA-bearing *A. pskemense* chromosome. The smallest chromosome carrying 5S rDNA in triploid *Allium* possessed only one signal in the pericentromeric region of the short chromosome arm (Figure 2g, h, i). This chromosome might represent a truncated and/or rearranged chromosome 7 of *A. roylei* (Figure 2e, f).

**Figure 3:**
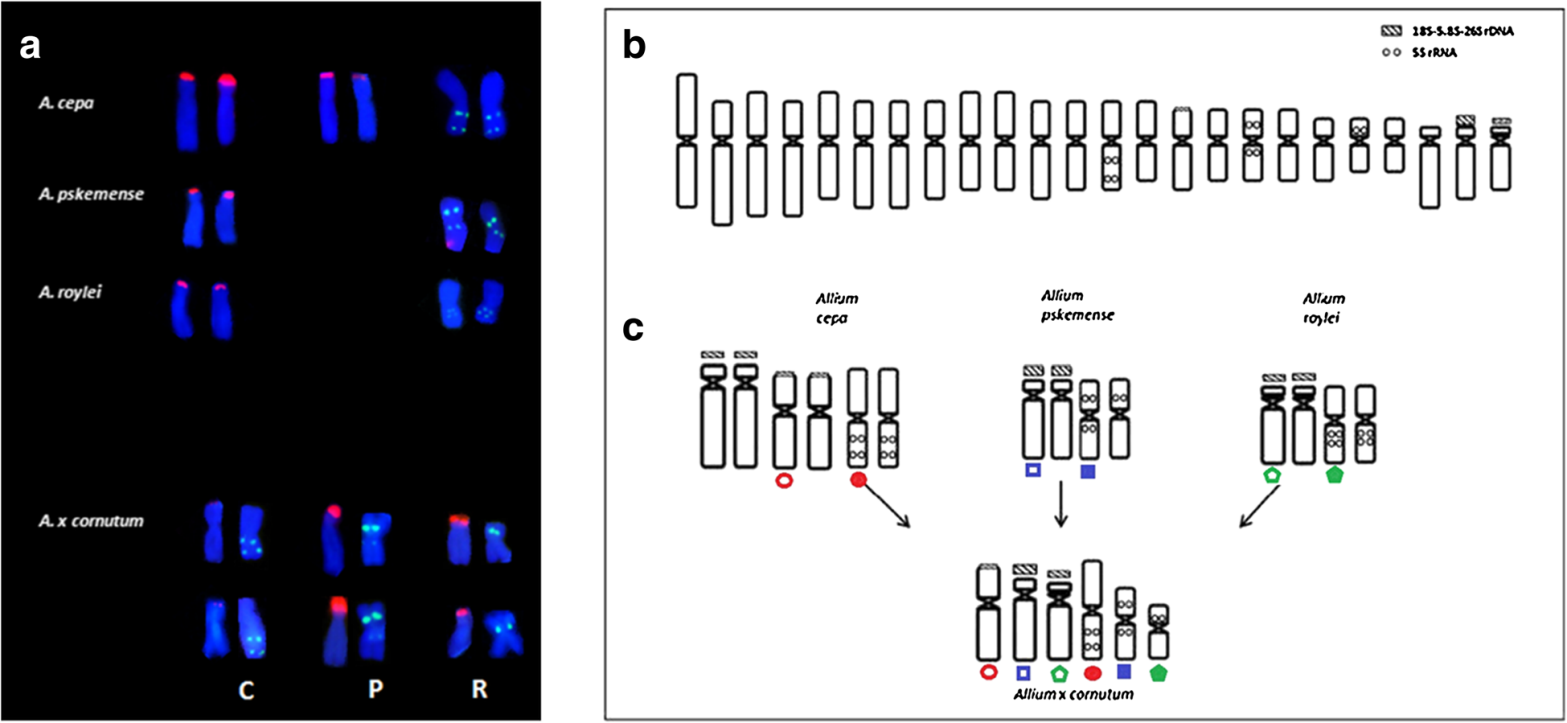
**Distribution and origin of rDNA loci in triploid *A*. × *cornutum*. (a)** Chromosomes carrying 5S and 35S rDNA in *A*. × *cornutum* and three putative parental taxa; **(b)** idiogram of *A*. × *cornutum* (modified from Puizina et. al. 1999); **(c)** origin and localisation of the 5S and 35S rRNA genes in triploid onion. Tri-colour circles, squares and pentagons were used to label the chromosomes that carry 5S and 35S rDNA in the progenitor species and the corresponding chromosomes in the triploid hybrid *A*. × *cornutum.*

The results of FISH mapping of 5S rDNA supported the inferences of the phylogenetic analyses of the 5S and 35S rDNA of triparental origin of *A. × cornutum* triploids. The three chromosomes carrying 5S rDNA genes in *A*. × *cornutum* likely originated from three different diploid *Allium* species (Figure 3).

**Figure 4:**
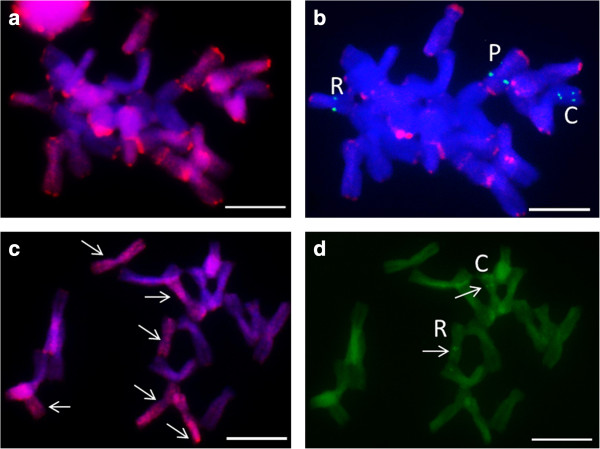
**Genomic *in situ *hybridisation (GISH); a, c) and subsequent 5S rDNA mapping (b, d) to mitotic metaphase chromosomes of *Allium × cornutum*. (a)** GISH with genomic DNA of *A. pskemense* (red) and *A. cepa* as blocking DNA, incomplete metaphase plate; **(b)** 5S rDNA (green) localisation in the same chromosomal spread **(c)** GISH with genomic DNA of *A. roylei* (red) and *A. cepa* as blocking DNA, incomplete metaphase plate; **(d)** 5S rDNA (green) mapping in the same chromosomal spread. The letters C, R, and P indicate chromosomes carrying the 5S signal and belonging to the three different genomes (due to insufficient washing of the genomic probe, red subtelomeric signals **(b)** remained visible in majority of the chromosomes). Scale bar = 10 μm. A few of the nuclei (top left and right corners) visible in **(a)** were lost during reprobing and are not visible in **(b)**.

**Figure 5:**
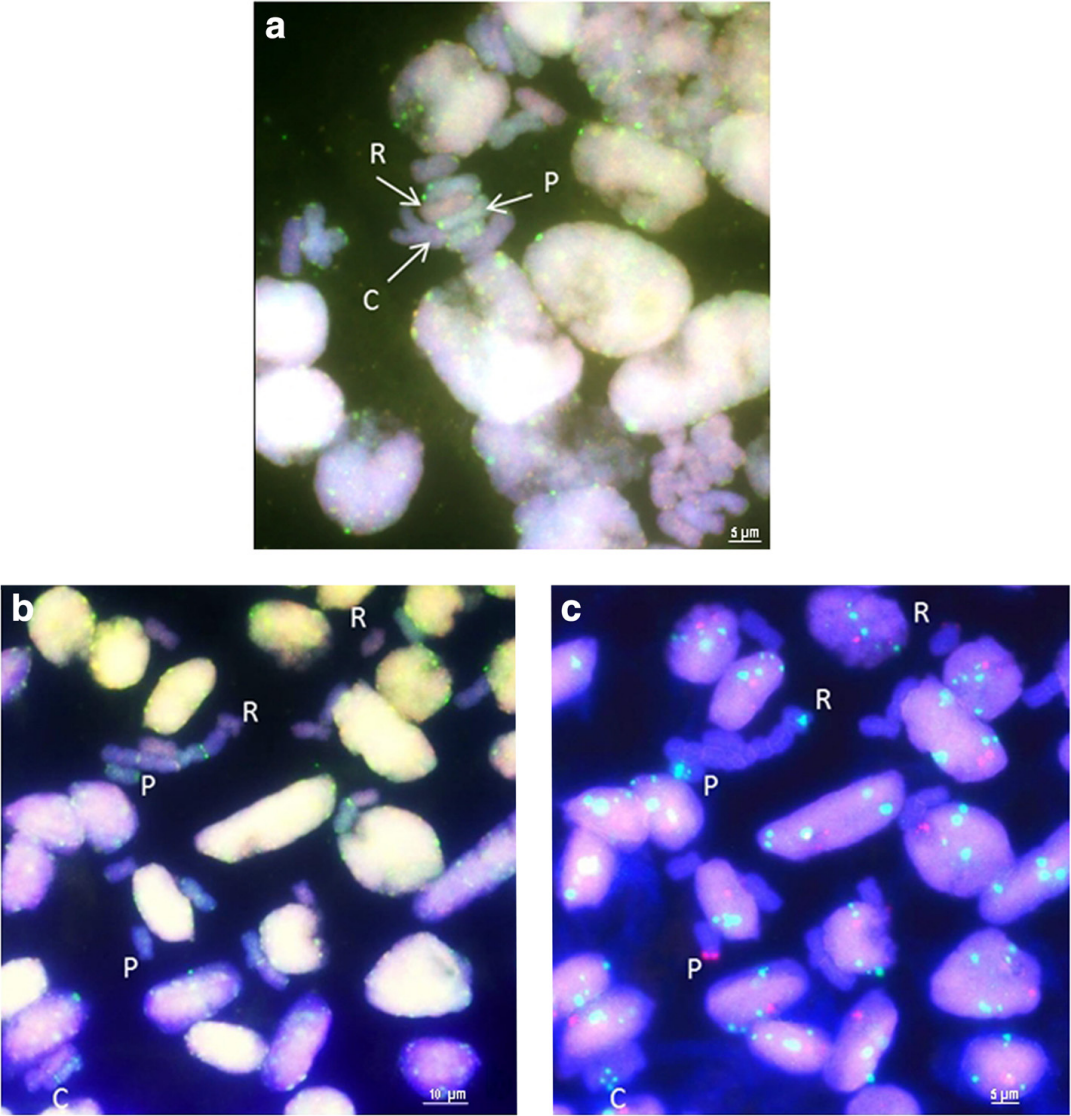
**Genomic *in situ *hybridisation (GISH) in *Allium × cornutum*; a, b) and subsequent 5S rDNA mapping (c) to mitotic metaphase chromosomes of *Allium × cornutum. *(a, b)** GISH with genomic DNA of *A. pskemense* (green), *A. roylei* (red) and *A. cepa* as blocking DNA (DAPI-blue); **(a)** Arrows and letters (C, R, and P) indicate the putative parental origins of the three genomes *(A. cepa, A. roylei* and *A. pskemense*, respectively); **(b, c)** The letters C, R, and P indicate chromosomes carrying 5S (green) and 35S (red) signals that belong to the three different parental genomes. C genome The 35S rDNA-carrying chromosome could not be identified; therefore it is not indicated. One nucleus (top left corner) visible in **(b)** was lost during reprobing and is not visible in **(c)**.

A combination of genomic *in situ* hybridisation (GISH) and FISH was attempted to identify the genomic origin of 5S rDNA-bearing chromosomes (Figures 4a–d; 5b, c). A single chromosome carrying two 5S rDNA loci (designated as P) was clearly labelled with *A. pskemense* genomic DNA, in contrast to the two other chromosomes carrying the 5S rDNA signals (designated as R and C), which remained unlabelled or were only weakly labelled (Figure 4b)*.* The hybridisation of the genomic DNA of *A. roylei* to the incomplete metaphase plate of *A.* × *cornutum* in the presence of an excess of unlabelled *A. cepa* total genomic DNA as blocking DNA (Figure 4c) allowed the labelling of six chromosomes of the triploid onion, which are marked by arrows. Among the chromosomes labelled with *A. roylei* genomic DNA, one chromosome carrying 5S rDNA is indicated (designated as R; Figure 4d). This chromosome corresponds to the smallest 5S rDNA-bearing chromosome, which is likely a truncated *A. roylei*-originating chromosome (Figure 3). With the aim to simultaneously discriminate between the three genomes of *A. × cornutum* (the putative C, R and P genomes), we performed genomic *in situ* hybridisation with two labelled parental DNA sequences as probes (*A. pskemense* genomic DNA was labelled in green; *A. roylei* genomic DNA was labelled in red), whereas the genomic DNA of *A. cepa* remained unlabelled and was used as blocking DNA (Figure 5a, b). Eight chromosomes were labelled predominantly with *A. pskemense* genomic DNA (P-genome; green; Figure 5a); furthermore, eight chromosomes hybridised predominantly with *A. roylei* genomic DNA (R-genome; red), and eight chromosomes remained nearly unlabelled (C-genome, blue). The chromosomes have subsequently been reprobed with 35S and 5S rDNA probes. A comparison of Figure 5b and c confirms that the three different 5S-bearing chromosomes of *A.* × *cornutum* belong to three different parental genomes.

## Discussion

### Triparental allopolyploid origin of *A.* × *cornutum* and the identification of its putative parental species

Two contradictory studies have been published concerning the origin of the triploid onion *A.* × *cornutum* [6,11; reviewed in 12]. The suggested triparental origin of a triploid has been proposed, but only two putative parental taxa were suggested (*A. cepa* and *A. roylei*), with the third parental taxon remaining unknown (the so-called “X genome”; [6]). The present study provides phylogenetic evidence for a triparental hybrid origin of *A*. × *cornutum*, supported by the mapping of the 5S and 35S rRNA genes in the chromosomes of putative parental taxa and the hybrid. Additionally, the third putative parental species, the wild Asian species *A. pskemense* B. Fedtsh., has been identified. These data support the complex hybrid origin of *A. × cornutum* and allow the rejection of previous hypotheses [11], which postulated the origin of *A*. × *cornutum* as a derivative of *A. cepa*. The current study clearly demonstrates that *A*. × *cornutum* contains three types of both ITS and NTS sequences, each grouping with one of three putative parental taxa, *Allium pskemense*, *A. roylei*, and *A. cepa*.

GISH allowed the detection of the three parental genomes in the hybrid, despite some level of cross-hybridisation. Using a combination of GISH and FISH, 5S and 35S rDNA-bearing chromosomes of the hybrid were shown to originate from the respective chromosomes of the putative parents.

### ITS sequence variability and 35S rDNA mapping

The phylogenetic analysis of the ITS region of *A.* × *cornutum* revealed three major ITS types denoted as P, R, and C. These types were recovered as separate clades in combined analyses and were shown to bear high sequence similarity to three diploid *Allium* species/lineages: *A. pskemense*, *A. roylei*, and *A. cepa*/*A. vavilovii. A. cepa* and *A. vavilovii* are closely related, with *A. cepa* being known only as a cultivated taxon; *A. vavilovii* was inferred as its closest wild relative [22].

Concordantly, three 35S rDNA loci were detected on three different chromosomes in triploid onion, in agreement with a previous report [52], and could be assigned to the three putative parental genomes. Our previous GISH analysis [6] showed that the medium-sized NOR chromosome carrying the largest 35S rDNA locus did not hybridise either with genomic probes of *A. cepa* or of *A. roylei*, thus being assigned to the unidentified X genome. The current study showed that this locus originates from *A. pskemense* and was the source of the majority of the cloned ITS sequence regions (41 out of 48 ITS clones). The major 35S rDNA locus originating from the *A. cepa* genome has either been lost during the evolution of the triploid genome or contains only very few copies that are below the detection limit of FISH. The second (minor) *A. cepa* locus, which has been detected in the triploid hybrid on the medium-sized submetacentric chromosome, contains a very small copy number and might be in the process of being lost. The single ITS sequence of the C*-*type (clone Hvar 21) that was recovered from the triploid onion genome likely represents the *A. cepa* minor 35S rDNA locus*.*

Earlier analyses of the activity of the NOR regions in triploid onion using silver staining indicated that all three 35S rDNA loci were active with a maximum of three nucleoli detected in the interphase nuclei of all five Croatian clones of *A*. × *cornutum*[14]. In contrast, Pran, the Indian clone of triploid *A. × cornutum,* possessed only a single active NOR on a medium–sized satellite chromosome [14]. Such a result indicates ongoing evolution of the rDNA in triploid onions over the whole species distribution range. Multiple origins of this triploid hybrid taxon are currently excluded based on the unique genome size, isozyme, RAPD and RFLP patterns of Pran, Ljutika and other analysed clones of triploid onion [6,10,11].

### NTS sequence variation and 5S rDNA mapping

The divergence of the NTS sequences in several well-documented allopolyploid systems proved very useful for the identification of the putative parent species: i.e., *Nicotiana tabacum*[48], *Zingeria*[53], *Anemone multifida* and *A. baldensis*[47], and *Melampodium*[1]. The 5S rDNA NTS sequences of *A.* × *cornutum* clustered into the three main clades (C, P, and R), which had high sequence homology to the three putative parental species *A. cepa*, *A. pskemense*, and *A. roylei*, respectively. Whereas the clades C (*A. cepa*) and R (*A. roylei*) were well supported, the clade P (*A. pskemense*) failed to form a single well-supported clade with its closest relatives, *A. pskemense, A. altaicum*, and *A.* × *proliferum.* This result could have been caused by significant intra-individual variability within the NTS region [28] as well as possible high genetic variation of 5S rDNA within *A. pskemense* and its relatives across their wide geographical distribution.

The results of the NTS sequence analysis were largely congruent with the cytogenetic mapping of 5S rDNA in *A.* × *cornutum,* and 5S rDNA loci were detected in different positions in three chromosomes of different morphology. The two larger 5S rDNA-bearing chromosomes greatly resembled the putative parental chromosomes of *A. cepa* and *A. pskemense.* The 5S rDNA-bearing chromosome having 5S rDNA loci distributed in a similar manner as in *A. pskemense* has also been labelled with *A. pskemense* genomic DNA in a GISH experiment. The smallest 5S rDNA-bearing chromosome of *A. × cornutum* has been shown to hybridise with genomic DNA of *A. roylei* ([6]; current study), thus confirming its origin from the R genome (*A. roylei*). This chromosome, however, differed from the parental 5S rDNA-bearing chromosome of *A. roylei* and possessed only a single 5S rDNA locus in the pericentromeric region of the short arm of the chromosome instead of two loci. At least two different scenarios can account for the observed truncation: i) the smallest 5S rDNA-bearing chromosome and the entire R genome could have originated from a diploid ancestor closely related to *A. roylei*, which is characterised by smaller chromosomes and only a single 5S rDNA locus; and ii) chromosomes of *A*. × *cornutum* originating from the R genome have undergone rearrangements after hybridisation and lost one of the 5S rDNA signals in the process. Based on our current understanding of genome restructuring after allopolyploidisation and a general trend of diploidisation of both the 5S and 35S rDNA loci, the second hypothesis is more likely [43,54-57], especially because the sequences of *A.* × *cornutum* in clade “R” are very similar to those of *A. roylei*. The triploid genome of *A*. × *cornutum* might have been subjected to additional genomic rearrangements such as inter-chromosomal translocation, deletions, and/or inversions.

### FISH mapping of 35S and 5S rRNA genes in *A. pskemense* and *A. roylei* and inferences of the phylogenetic relationships in section *Cepa*

Although the phylogenetic relationships among 12 species of sections of *Cepa* have been inferred from analyses of plastid regions and ITS [20,22], the chromosomal positions of 35S and 5S rDNA have so far been determined for only three species: *A. cepa*, *A. fistulosum* and *A. altaicum*[58-61]. The two 5S rDNA loci in *A. cepa* were located on the longer arm of chromosome 7 (as confirmed by our data), whereas in *A. fistulosum* a single 5S rDNA locus was detected interstitially in the short arm of chromosome 7. In natural diploid homoploid hybrids of *A. cepa* and *A. fistulosm*, top onions (*Allium* × *proliferum* (Moench) Schrad. and *Allium wakegi* Araki (both 2*n* = 16), the two chromosomes carrying the 5S rDNA signals corresponded to the 5S rDNA-bearing chromosomes of the parental species [58]. In this study, 35S and 5S ribosomal genes were mapped for the first time in somatic chromosomes of diploid *A. pskemense* and *A. roylei*. These two species possessed an identical number of 35S rDNA loci but differed in the positions of the 5S rDNA loci on chromosome 7. The localisation of 5S rDNA in *A. roylei* chromosome 7 resembles more closely that of *A. cepa*, whereas *A. pskemense* is more similar to *A. fistulosum* and *A. altaicum.* This finding supports the hypothesis of Son *et al.*[28] that *A. pskemense* is more closely related *to A. fistulosum* and *A. altaicum.* The number and position of the 5S rDNA loci, therefore, proved to be evolutionarily informative in analysing the species of *Allium* from section *Cepa*.

## Conclusions

The combined molecular phylogenetic and cytogenetic data obtained in this study provide evidence for a unique triparental origin of triploid onion *A.* × *cornutum* and identified all three putative diploid parental species, *A. cepa*, *A. pskemense*, and *A. roylei.* These results are in agreement with previously published data [6,9,14] and provide new and stronger evidence for the origin of the distinct and complex odd-ploidy allopolyploid *A*. × *cornutum*. The sequence of events leading to the origin of the triploid onion and its phylogeography cannot yet be elucidated and will be addressed using other molecular approaches.

## Methods

### Plant materials and DNA extraction

Four clones of *A.* × *cornutum* (known in Croatia under the name Ljutika) were obtained from local gardens and vineyards at four well-separated localities of the Croatian seaside region (Dubrava and Kaštela) and islands (Vis, Hvar). *A. pskemense* B. Fedt. (CGN21442) and *A. roylei* Stearn (CGN20520) seeds were kindly provided by the Centre for Plant Breeding and Reproduction Research, Wageningen, The Netherlands. The commercial cultivar *A. cepa* cv, ‘Holland Yellow’ was used to obtain the DNA and chromosome complements of *A. cepa* Genomic DNA was extracted from young leaves using the CTAB method according to Saghai Maaroof *et al.*[62].

### PCR amplification and cloning

The ITS1-5.8S-ITS2 region of 35S rDNA was amplified by PCR using the universal primers ITS1 and ITS4 and the procedures described by Bezić *et al.*[63]. The whole coding and non-transcribed spacer (NTS) region of the 5S rDNA gene was amplified using the primers and conditions from Weiss-Schneeweiss *et al.*[57]. The amplified products were visualised and confirmed by 1% agarose gel electrophoresis, extracted from the gel, ligated into pGEM-T Easy vectors (Promega, Madison, Wisconsin, USA) and cloned into competent JM109 *E. coli* cells. DNA from individual plasmids carrying inserts was isolated using a Plasmid Mini Kit (Qiagen, Hilden, Germany). Purified plasmid DNA was sent to Macrogen (Seoul, Korea) for sequencing of the inserts.

### Sequence analysis

The DNA sequences were assembled and prealigned using BioEdit ver. 7.0.5.3 [64]. They were then aligned in ClustalW [65] and implemented in MEGA5 [66], and the alignment was refined manually. The sequences were deposited in GenBank (Table 1). To avoid multiple submissions of identical sequences, we sent only one sequence of each type. To infer the phylogenetic relationships from the newly obtained ITS and NTS sequences of *A. × cornutum* and other closely related *Allium* species, the sequences were subjected to a similarity search against the non-redundant nucleotide sequence database using the NCBI (National Centre for Biotechnology Information) BLASTN network service. Sequence alignments of newly amplified regions and sequences of other related *Allium* species deposited in GenBank were performed using MEGA5 [66]. Polymorphic and variable sites as well as different haplotypes were generated using DnaSP Ver. 5.10 [67]. A Bayesian analysis was performed with MrBayes 3.1 [68] with 4 chains of 1,000,000 generations, trees sampled every 100 generations and the burn-in value set to 25% of the sampled trees. The best-fit substitution model was used as determined by the Akaike Information Criterion [69] as implemented in jModelTest 0.1.1 [70]. A maximum-likelihood analysis using starting trees obtained by neighbour-joining and TBR branch swapping with model parameters was performed using PAUP* 4.0b10 [71]. The number of bootstrap replicates was set to 1000. Phylogenetic trees were displayed in FigTree v1.3.1.

### Chromosome preparation, fluorescence *in situ* hybridisation (FISH) and genomic *in situ* hybridisation (GISH)

Chromosomes for FISH and GISH were prepared as described by Puizina *et al.*[6]. Clone pTa794 contained the complete 410-bp BamHI fragment of the 5S rRNA gene, and the spacer region of wheat [72] was used as the 5S rDNA probe. The 2.4 kb *Hind*III fragment of the partial 18S rRNA gene and ITS1 from *Cucurbita pepo*, cloned into pUC19 [73], were used as the 18S rDNA probe. The 5S rDNA probe was labelled with digoxygenin using a DIG-nick translation kit (Roche Diagnostics, Mannheim, Germany), whereas 18S rDNA was labelled with biotin using a BIO-nick translation kit (Roche Diagnostics, Mannheim, Germany). The genomic DNA (1 μg/reaction) was labelled with biotin using a BIO-nick translation kit (Roche Diagnostics, Mannheim, Germany) according to the supplier’s instructions.

The FISH method followed the procedures outlined in Weiss-Schneeweiss *et al.*[57,74]. Briefly, the preparations were re-fixed and air dried, and chromosomal DNA was denatured in 70% (v/v) deionised formamide in 2× SSC, pH 7.0 at 70°C for 2 min, dehydrated through an ethanol series and air-dried. The hybridisation mixture containing labelled probes (100–150 ng/slide), 20–100x excess blocking DNA (for GISH) or salmon sperm DNA (for FISH), 50% formamide, 2x SSC, 10% dextran sulphate, and 0.15% sodium dodecyl sulphate (SDS) was denatured at 75°C for 10 min. The probe was applied to the slides and incubated at 37°C in a humid chamber overnight. Subsequently, the slides were washed for 5 min in 2x SSC at 39°C, for 5 min in 0.1x SSC at 39°C, and for 5 min in 2x SSC + 0.2% Tween 20 at 39°C. Biotin- and digoxygenin-labelled probes were detected using Extravidin-Cy3 (2.5 μg/mL) and anti-digoxygenin-FITC (5 μg/mL), respectively, both in 2% BSA in 2x SSC + 0.2% Tween 20 buffer at 37°C for 60 min. The slides were subsequently washed twice for 7 min in 2x SSC at 42°C and for 7 min in 2x SSC + 0.2% Tween 20 at 42°C. Finally, they were mounted in 20 μL of the antifade solution Vectashield containing 0.5 μg/mL DAPI (Vector Laboratories, Burlingame, CA, USA) and stored at 4°C. The slides were examined with a Zeiss Axioimager M1 epifluorescence microscope with a high-resolution microscopy camera (Carl Zeiss AxioCam MR Rev3) using Axio Vision Rel. 4.7 software (Karl Zeiss, Vienna, Austria). For rDNA localisation, an average of 15–20 metaphase plates were analysed for each species. GISH hybridisation and detection were performed using the same protocols.

## Competing interests

The authors declare no competing interests.

## Authors’ contributions

The experimental design was conceived by JP and HWS. The experiments were performed by ZF, IS, JK, and TY. The data were analysed by JP with assistance from HWS and IS. This paper was written by JP, HWS and ZF. All authors read and approved the final manuscript.

## Supplementary Material

Additional file 1: Figure S1 Sequence variation in the nuclear internal transcribed spacer (ITS) from four different plants (clones) of *A. × cornutum* and its parental species, *A. pskemense*, *A. roylei* and *A. cepa*. Click here for file

Additional file 2: Figure S2 Sequence variation in the non-transcribed spacer (NTS) of the 5S rDNA region in three different plants (clones) of *A.* × *cornutum*. Click here for file
