# Supplementary figures and images for: Triparental origin of triploid onion, Allium × cornutum (Clementi ex Visiani, 1842), as evidenced by molecular, phylogenetic and cytogenetic analyses
Source: BMC Plant Biol. 2014 Jan 13;14:24. doi: 10.1186/1471-2229-14-24 (PMC3899691; doi:10.1186/1471-2229-14-24)

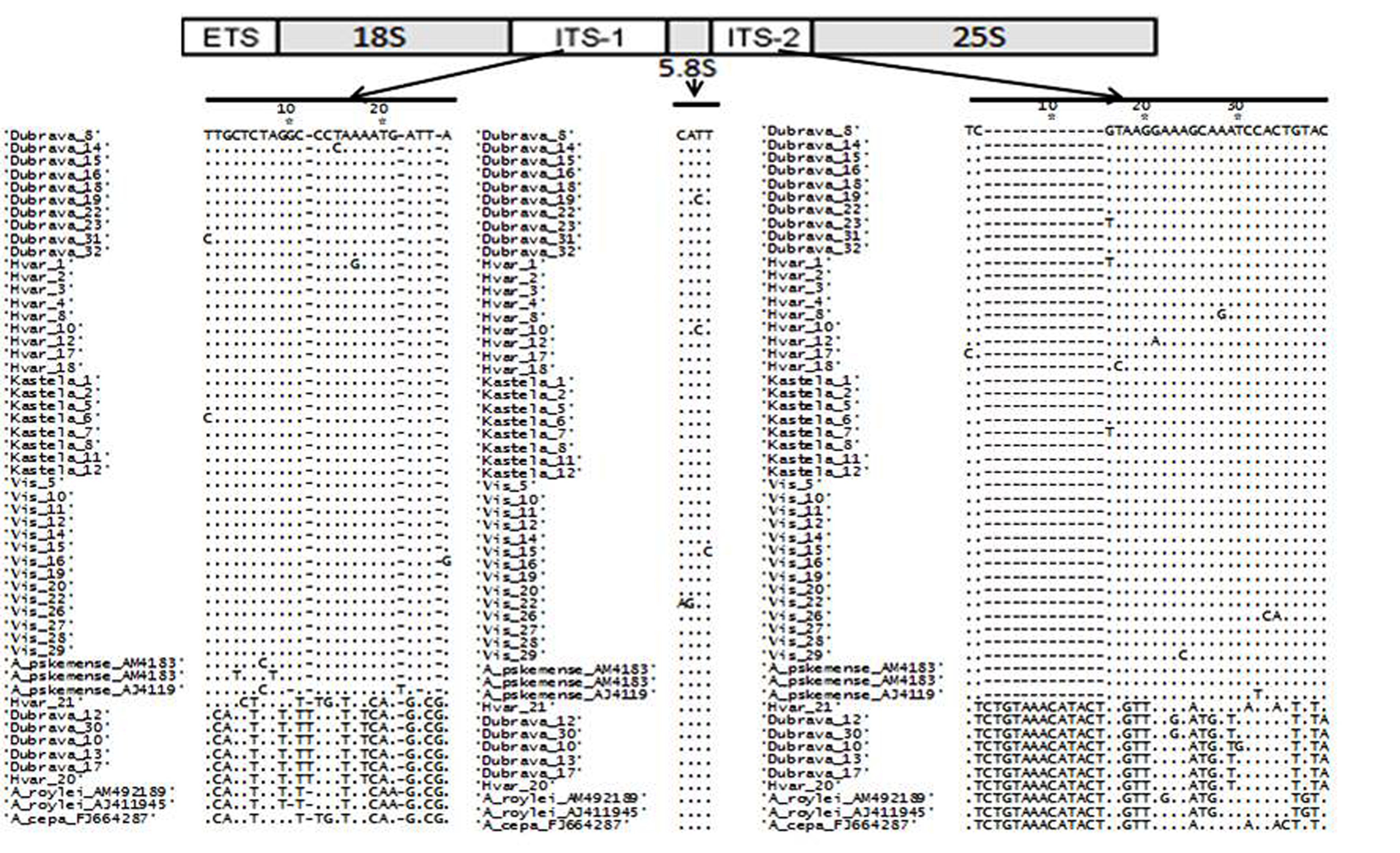

Supplement: Additional file 1: Figure S1 — Sequence variation in the nuclear internal transcribed spacer (ITS) from four different plants (clones) of A. × cornutum and its parental species, A. pskemense, A. roylei and A. cepa. [file 1471-2229-14-24-S1.tiff]

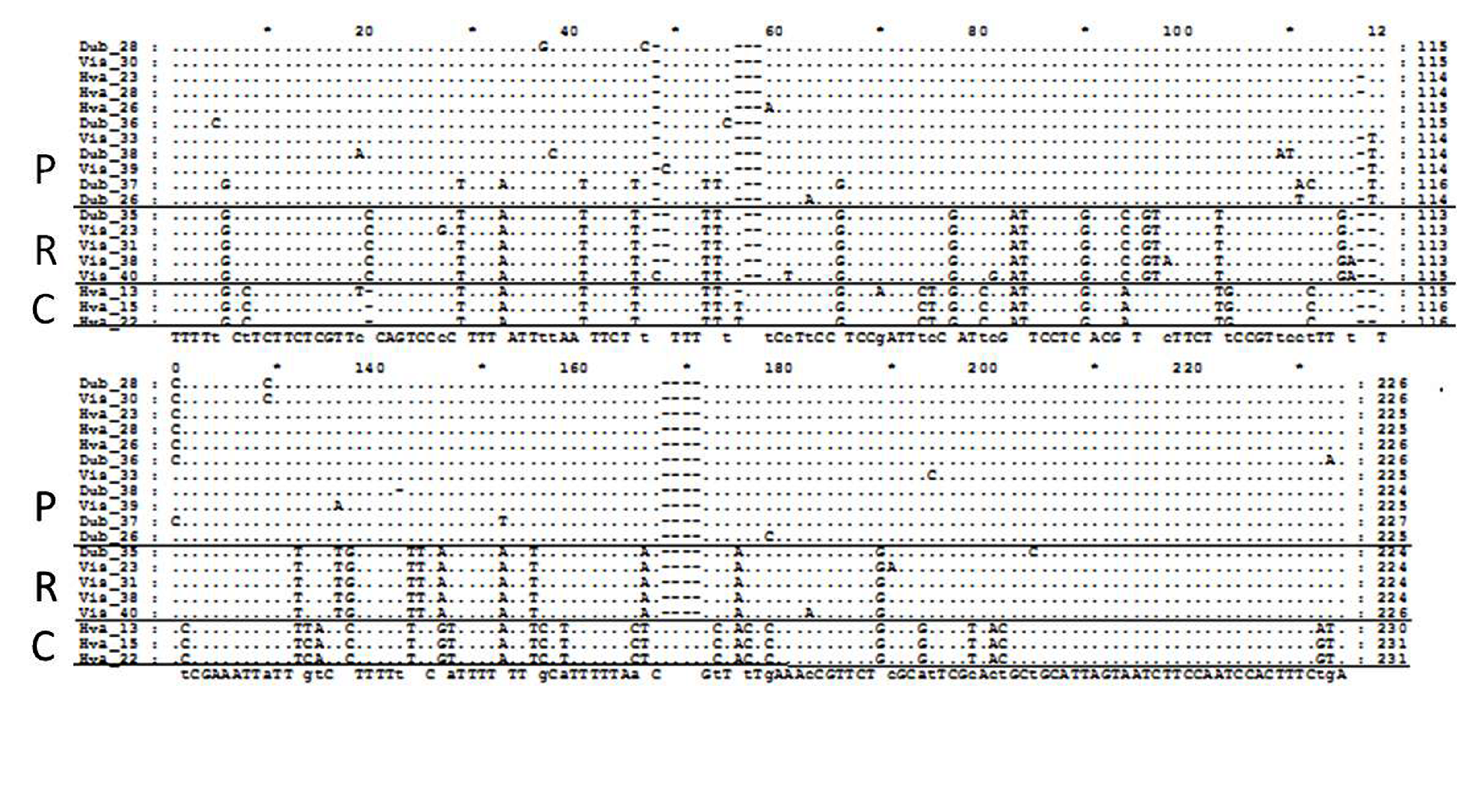

Supplement: Additional file 2: Figure S2 — Sequence variation in the non-transcribed spacer (NTS) of the 5S rDNA region in three different plants (clones) of A. × cornutum. [file 1471-2229-14-24-S2.tiff]
